# Trends in *Neisseria meningitidis* serogroups amongst patients with suspected cerebrospinal meningitis in the meningitis belt of Ghana: a 5-year retrospective study

**DOI:** 10.1186/s12879-023-08196-x

**Published:** 2023-04-06

**Authors:** Stebleson Azure, Abass Abdul-Karim, Braimah Baba Abubakari, John B. Eleeza, Daron Davies A. Agboyie, Enoch Weikem Weyori, Jun Yong Choi

**Affiliations:** 1 Yonsei University Graduate School of Public Health, Seoul, Korea; 2 Tamale Public Health Laboratory, Tamale, Ghana; 3 Northern Regional Health Directorate, Tamale, Ghana; 4 Department of Internal Medicine, Yonsei University College of Medicine, 50-1 Yonsei-Ro, Seodaemun-Gu, Seoul, 03722 South Korea

**Keywords:** Conjugate, Meningococcal, Non-groupable, Polysaccharide, Polymerase chain reaction, Vaccination, Ghana Meningitis belt, Retrospective study, *Neisseria meningitidis* serogroups, 2016 to 2020

## Abstract

**Background:**

Serogroup A *Neisseria meningitidis* was the major cause of meningococcal meningitis epidemics in the African meningitis belt before 2010 when the monovalent meningococcal A conjugate vaccine (MenAfriVac) was introduced in the region. Therefore, this study aimed to establish the trends in *N. meningitidis* serogroups from 2016 to 2020 in Ghana’s meningitis belt.

**Methods:**

Polymerase chain reaction (PCR) confirmed laboratory results of suspected cases of cerebrospinal meningitis from January, 2016 to March, 2020 were obtained from the Tamale Public Health Laboratory. The data were subjected to trend analysis using Statistical Package for the Social Sciences version 25. Differences between discrete variables were analyzed using the Cochran–Armitage trend test.

**Results:**

Of the 2,426 suspected cases, 395 (16.3%) were confirmed positive for *N. meningitidis* using PCR. Serogroup X showed a significant upward trend (*P* < 0.01), and serogroup W showed a downward trend (*P* < 0.01). However, no significant trend was observed for any other serogroup.

**Conclusion:**

This study showed the emergence of serogroup X, a non-vaccine type, as the predominant *N. meningitidis* serogroup in the wake of a declining serogroup W in Ghana’s meningitis belt.

**Supplementary Information:**

The online version contains supplementary material available at 10.1186/s12879-023-08196-x.

## Background

*Neisseria meningitidis* is one of the most common causes of meningitis worldwide. Large epidemics of the disease due to meningococci have spread during the last decade throughout a large area of Africa’s ‘meningitis belt’ and outside [1]. Cerebrospinal meningitis (CSM) caused by *N. meningitidis* is a contagious disease. The first clear description of the symptoms of the disease was given by Viesseux following its classical epidemic in Geneva [2]. Epidemics of meningitis due to *N. meningitidis* are a serious medical emergency with both public health and socioeconomic implications. *N. meningitidis* was first identified in 1884 [3] and isolated from patients with CSM by Weichsel Baum in Vienna in 1887 as a gram-negative diplococcus. Based on the antigenicity of its capsular polysaccharides, it was classified into 12 serogroups, with most invasive cases caused by serogroups A, B, C, X, Y, and W [4]. Traditionally, serogroups Y and W occasionally caused diseases; however, since 2000, outbreaks and epidemics have been the result of serogroup W [5–8]. Epidemics of meningitis due to *N. meningitidis* are often difficult to predict, leading to delayed initiation of control measures, such as immunisation, resulting in poor outcomes.

According to the World Health Organization, an estimated 500,000 cases and 50,000 deaths annually worldwide are associated with *N. meningitidis,* with children and young adults being the most vulnerable [9]. Serogroup A meningococcus is implicated in a significant number of epidemics of meningitis that occur in the African meningitis belt and China but rarely in industrialised countries. Serogroup C has also been implicated in disease outbreaks and epidemics [10, 11]. Serogroups B and C were responsible for endemic meningococcal meningitis with occasional occurrences as a result of serogroups W, Y, and X [12, 13]. However, serogroup W also causes endemic diseases in some African meningitis belt countries. Serogroup A epidemics in Africa’s meningitis belt occur every 8–12 years, with each wave following a gradual increase in volume-decrease in the volume of cases pattern [14]. Similar to other bacterial meningitis, the incidence of meningococcal meningitis is seasonally dependent, with peaks during the dry season (December–May) and decreases rapidly, even in times of major epidemics with the start of the rainy season [14, 15].

Although meningococcal meningitis is endemic in various regions worldwide, its burden is remarkable in 26 countries comprising the ‘meningitis belt’ of subSaharan Africa, stretching from Senegal in the west to Ethiopia in the east [16]. Meningococcal meningitis is hyperendemic in the region, with the number of cases approaching 1,000 per 100,000 inhabitants per year during the dry season. The annual epidemics in this region are broadly distributed across age groups. However, sporadic cases occur mostly in young children [9].

Serogroup A was the main cause of most cases of CSM in the meningitis belt of subSaharan Africa between 1993 and 2012. It accounts for 80% of the epidemics, with approximately 1 million cases and 100,000 deaths [16]. Consequently, a monovalent meningococcal A conjugate vaccine (MenAfriVac) was developed and prequalified for use in children and adults aged 1–29 years [17, 18]. It was used in a mass vaccination campaign in Burkina Faso, Mali, and Niger in 2010 [19]. As a result of vaccine introduction, by 2014, there were no cases due to serogroup A in these three countries [20] and other African countries, including Ghana, which introduced the vaccine into their immunisation program. The decline in meningitis due to serogroup A resulted in an increase in the number of serogroup W cases, making it the predominant aetiological agent responsible for 55% of the confirmed cases in 2012 in the region [21]. Meningitis cases due to serogroup C are also on an upward trajectory in the subSaharan African region. Up to 82.7% of the 433 confirmed cases during the outbreak in Nigeria from December, 2016 to June, 2017 were due to *N. meningitidis* serogroup C. It is considered the largest outbreak of meningococcal meningitis due to serogroup C worldwide [22].

The northern part of Ghana lies within Africa’s ‘meningitis belt’ and has been experiencing bacterial meningitis outbreaks during the dry seasons, usually from December to May each year. Although only the northern part of Ghana is at high risk of meningitis outbreaks, cases have been reported in the Ashanti region in 2016. There were also reported cases in the central and Ashanti regions between December, 2019 and April, 2020.

**Fig. 1:**
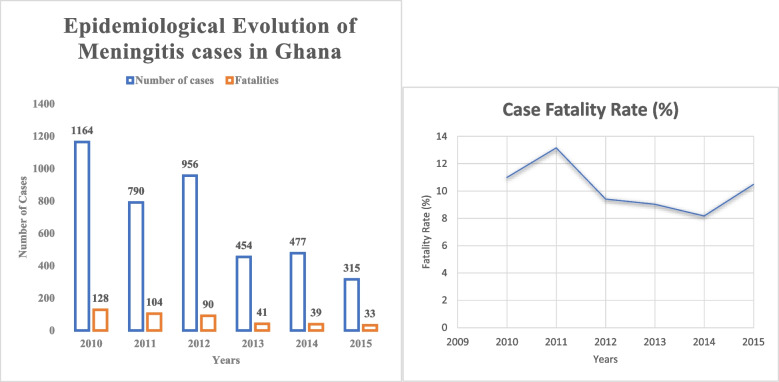
Epidemiological evolutions of meningitis cases and case fatality rate in Ghana (2010–2015)

Figure 1 illustrates the epidemiological evolution of meningitis cases and fatality rates from 2010 to 2015 in Ghana [23]. However, it should be noted that until 2012, cases were not confirmed using real-time polymerase chain reaction (PCR). Testing was mainly performed using gram staining, latex agglutination, culture, and antimicrobial sensitivity testing.

Despite the interventions put in place by the Ministry of Health to reduce the burden of bacterial meningitis in Ghana, annual outbreaks still occur. These outbreaks are mainly caused by *Streptococcus pneumoniae* and *N. meningitidis*.

With the introduction of real-time PCR at the Zonal Tamale Public Health Reference Laboratory in 2012, the aetiological agents of bacterial meningitis have been profiled in detail. However, the actual trends in the nongroupable (NG) and nonvaccine type serogroups of *N. meningitidis* and the impact of vaccination programs on vaccine-preventable serogroups have not been adequately highlighted. Therefore, it is imperative that a study of this nature is carried out to help establish the trends in the various serogroups of *N. meningitidis* in the country’s meningitis belt to determine whether there was an emerging threat from any of either the NG or nonvaccine type *N. meningitidis* serogroups, hitherto not posed any serious threat in the past. In addition, it is important to establish the influence of vaccination programs on the trends of vaccine-preventable serogroups.

Knowledge of the trends in the various serogroups of *N. meningitidis* is crucial to form targeted public health interventions to control the situation. It would also inform the need to undertake a molecular study of the circulating strains to determine if the upward trend is due to mutation of the original strain, based on which vaccines were developed. If the upward trend is found to be in the nonvaccine type, it would inform the need for the appropriate authorities to start taking the necessary steps to educate the at-risk population and take the necessary steps toward the development of vaccines targeted at the particular serogroup to bring it under control before it becomes a public health threat. Moreover, Ghana can group only 6 of the 12 known serogroups of *N. meningitidis*. However, occasionally, some unknown or NG serogroups have been isolated. If an upward trend is seen in the NG type, it would inform the need for further studies to identify those serogroups so that logistics are procured for effective surveillance.

Therefore, the primary objective of this study was to establish the trends in *N. meningitidis* serogroups over 5 years in Ghana’s meningitis belt from 2016 to 2020.

## Materials and method

### Study area

The study data was collected at the Zonal Public Health Laboratory in Tamale, situated in the northern region of Ghana. The Tamale Zonal Public Health Laboratory is designated as the National Reference Laboratory for the confirmatory diagnosis of meningitis caused by bacteria in Ghana. Tamale is located in the centre of the northern region, with an approximate land size of 647 km^2^ with a population of 371,351 inhabitants. Real-time PCR was used for the confirmation of bacterial meningitis.

### Study design and population

This retrospective study was conducted using laboratory results of patients with suspected CSM from January, 2016 to March, 2020, confirmed using PCR at the Tamale Public Health Laboratory. Data beyond March, 2020 were unavailable at the time of this study. The study population included suspected patients diagnosed with CSM in Ghana’s meningitis belt within the study period. Only data from suspected patients who were confirmed residents of Ghana at the time of the suspected disease condition were considered in the study.

The clinical definition of suspected CSM included sudden onset of fever (> 38.5 °C rectal or > 38.0 °C axillary), neck stiffness, altered consciousness, headache, petechial or purpuric rash for suspected patients aged 1 year and over, fever accompanied by bulging fontanelle, vomiting drowsiness, irritability, and seizures (with or without petechial rash) for patients aged under 1 year [24, 25].

The inclusion criteria were all PCR-confirmed cases of suspected meningitis within the study period and all *N. meningitidis-*positive cases with serogroup results. Patients with incomplete data, including demographic data, such as age, sex, region of residence, and *N. meningitides-*positive cases without serogroup results, were excluded from the study. A total of 2,426 patients suspected of having CSM within the study period were confirmed to be either negative or positive using real-time PCR, and all 395 cases confirmed to be *N. meningitidis* positive were included in this study.

**Fig. 2:**
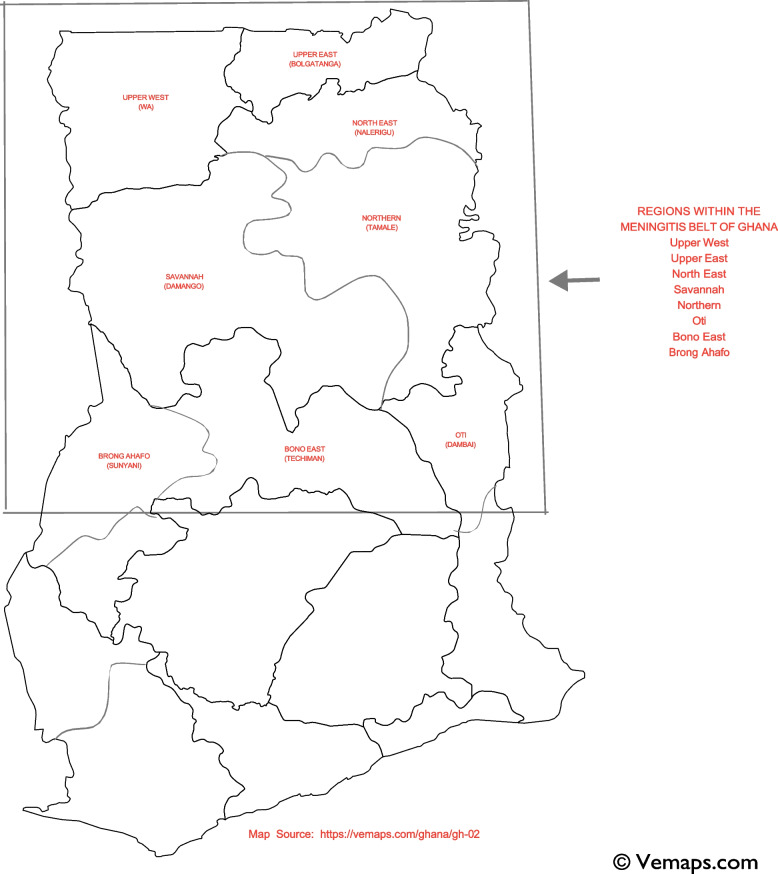
Map of Ghana indicating regions within the meningitis belt of the country

Below is a map of Ghana (Fig. 2), indicating the regions prone to CSM outbreaks.

### Data source, collection, and management

Patient demographic data was obtained from the case investigation forms accompanying cerebrospinal fluid (CSF) samples for laboratory confirmation. Patient demographic data included age, sex, and region of residence. The PCR-confirmed laboratory data collected included results for both speciation and serogroups of all *N. meningitides-*positive and speciation only for all other cases within the study period. The collected data was entered into a password-protected database with access only by the principal investigator.

### Ethical consideration

The Institutional Review Board (IRB) of the Yonsei University Health System approved this study (Y-2020–0105), and the need for informed consent was waived because of the retrospective nature of the study, and the data was anonymised (IRB Approval Certificate in S2 Text). Permission to use the data was also obtained from the Management of the Tamale Public Health Reference Laboratory. The names and epidemiological numbers were unlinked anonymously to ensure the confidentiality of patients prior to data access. All methods used in this study were carried out in accordance with relevant guidelines and regulations.

### Laboratory method: confirmation using real-time PCR

All samples were confirmed using direct real-time PCR for species identification. A triplex detection method was used for *S. pneumoniae* serotype identification using Cy5, HEX, and FAM as differentiating dyes. The serogroups of *N. meningitidis* and *Haemophilus influenzae* were identified via monoplex detection using FAM and ROX dyes, with ROX as the reference dye. For triplex detection, a single master mix was prepared and used for the simultaneous detection of *N. meningitidis, S. pneumoniae,* and *H. influenzae* species. The constituents of the master mix included primers (both forward and reverse) and probes of all the species tested in equal volumes, PCR grade water, and Multiplex Quanta. The ratios were 12.5 µL:7.5 µL:1 µL for the master mix, PCR grade water, and primers and probes, respectively, for a sample [26]. The target genes for PCR detection were the Cu and Zn superoxide dismutase gene, *sodC*, autolysin gene (*lytA*), and protein D encoding gene, *hpd*, for *N. meningitidis, S. pneumoniae, and H. influenzae*, respectively.

All samples which tested positive for *N. meningitidis* were selected, and their serogroups were identified using the monoplex detection method. Serogroup identification using a slide agglutination procedure with polyclonal antisera was not considered because it is usually associated with nonspecific or cross-reactions [27]. A master mix was prepared for each of the six tested serogroups. The constituents of each master mix included the primers (both forward and reverse) and probes of the serogroup of interest, PCR-grade water, and a monoplex Quanta with low ROX in the same ratios as for the triplex detection. Reaction templates were prepared for each reaction depending on the number of samples tested, and the master mix was prepared accordingly. The master mix and samples were added to the PCR reaction plate wells at a ratio of 23 µL:2 µL, respectively [26]. The controls were run simultaneously with the samples. When new dilutions of primers and probes were prepared, they were controlled before testing the patient samples.

The prepared reaction plate with the sample reagent mix was loaded into the Agilent AriaMx Real-Time PCR analyser for amplification and detection at a 50-cycle time of 01:42:47. The cycling conditions included a first step at 95 °C for 15 s and a second step at 60 °C for 1 min for the amplification segment [26]. The amplification curves and quantitation cycle (cq) values for the samples at the end of the reaction cycles were analysed, and the results were interpreted. Samples with cq ≤ 34 were interpreted as positive; those between 34 and 35 were considered equivocal; and those with no cq or cq greater than 35 were considered negative [26].

### Statistical analysis

**Fig. 3:**
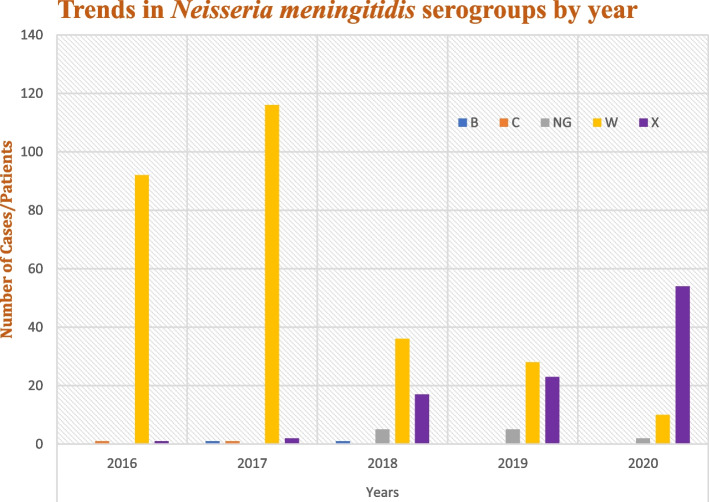
Distribution/trends of *Neisseria meningitidis* serogroups confirmed using polymerase chain reaction by year

The collected data was entered into the Statistical Package for Social Sciences version 25 software program and analysed. Differences between discrete variables were analysed using the Cochran–Armitage trend test. A *P*-value < 0.01 was considered statistically significant. The trends in serogroups are presented in tables (see Tables 1, 2, 3 and 4) and graphically as a bar chart (see Fig. 3).

**Table 1:** Sociodemographic distribution of the various serogroups of *Neisseria meningitides-*positive cases confirmed using polymerase chain reaction

	B *N* = 2	C *N* = 2	NG *N* = 12	W *N* = 282	X *N* = 97	Total *N* = 395
	N (%)	N (%)	N (%)	N (%)	N (%)	N (%)
**Sex**						
Male	1 (50.0)	2 (100.0)	7 (58.3)	156 (55.3)	57 (58.8)	223 (56.5)
Female	1 (50.0)	0 (0.0)	5 (41.7)	126 (44.7)	40 (41.2)	172 (43.5)
**Age Group**						
Under 1	0 (0.0)	0 (0.0)	0 (0.0)	8 (2.8)	0 (0.0)	8 (2.0)
1–4	0 (0.0)	0 (0.0)	1 (8.3)	58 (20.6)	9 (9.3)	68 (17.2)
5–10	0 (0.0)	1 (50.0)	4 (33.3)	82 (29.1)	44 (45.4)	131 (33.2)
11–15	1 (50.0)	0 (0.0)	4 (33.3)	50 (17.7)	23 (23.7)	78 (19.7)
16–23	1 (50.0)	1 (50.0)	1 (8.3)	56 (19.9)	15 (15.5)	74 (18.7)
24–44	0 (0.0)	0 (0.0)	2 (16.7)	18 (6.4)	3 (3.1)	23 (5.8)
45–64	(0.0)	0 (0.0)	0 (0.0)	10 (3.5)	3 (3.1)	13 (3.3)
Mean age (SD)	14.2 (± 13.4)	10.8 (± 8.9)	11.6 (± 9.0)	15.4 (± 12.9)	11.4 (± 8.7)	12.4 (± 10.8)
**Regions**						
Brong Ahafo	0 (0.0)	0 (0.0)	0 (0.0)	5 (1.8)	0 (0.0)	5 (1.3)
Northern	1 (50.0)	0 (0.0)	6 (50.0)	183 (64.9)	15 (15.5)	205 (51.9)
Upper East	0 (0.0)	0 (0.0)	3 (25.0)	40 (14.2)	40 (41.2)	83 (21.0)
Upper West	1 (50.0)	2 (100.0)	3 (25.0)	54 (19.1)	42 (43.3)	102 (25.8)

B, C, W, and X: *Neisseria meningitidis* serogroups B, C, W, and X, respectively

*NG* Nongroupable serogroups, *SD* Standard deviation

**Table 2:** Distribution/trend of the various *Neisseria meningitidis* serogroups confirmed using polymerase chain reaction by year

*N. meningitidis* Serogroups	2016 *N* = 94	2017 *N* = 120	2018 *N* = 59	2019 *N* = 56	2020 *N* = 66	Total *N* = 395	Cochran-Armitage *p*-value
	n (%)	n (%)	n (%)	n (%)	n (%)	n (%)	
B	0 (0.0)	1 (0.8)	1 (1.7)	0 (0.0)	0 (0.0)	2 (0.5)	0.8732
C	1 (1.1)	1 (0.8)	0 (0.0)	0 (0.0)	0 (0.0)	2 (0.5)	0.3303
NG	0 (0.0)	0 (0.0)	5 (8.5)	5 (8.9)	2 (3.0)	12 (3.0)	0.0334
W	92 (97.9)	116 (96.7)	36 (61.0)	28 (50.0)	10 (15.2)	282 (71.4)	< 0.01
X	1 (1.1)	2 (1.7)	17 (28.8)	23 (41.1)	54 (81.8)	97 (24.6)	< 0.01

B, C, W, and X: *Neisseria meningitidis* serogroups B, C, W, and X, respectively

*NG* Nongroupable serogroups

**Table 3:** Distribution/trend of the various *Neisseria meningitidis* serogroups confirmed using polymerase chain reaction by age group and year

		2016 *N* = 94	2017 *N* = 120	2018 *N* = 59	2019 *N* = 56	2020 *N* = 66	Total *N* = 395	Cochran-Armitage *P*-value
Age Group	Serogroup	N (%)	N (%)	N (%)	N (%)	N (%)	N (%)	
Under 1	W	2 (100.0)	2 (100.0)	3 (100.0)	1 (100.0)	0 (0.0)	8 (100.0)	0.99
1–4	NG	0 (0.0)	0 (0.0)	0 (0.0)	0 (0.0)	1 (11.1)	1 (1.5)	0.99
	W	21 (100.0)	25 (100.0)	6 (75.0)	5 (100.0)	1 (11.1)	58 (85.3)	0.99
	X	0 (0.0)	0 (0.0)	2 (25.0)	0 (0.0)	7 (77.8)	9 (13.2)	1.00
5–10	C	1 (4.2)	0 (0.0)	0 (0.0)	0 (0.0)	0 (0.0)	1 (0.8)	0.19
	NG	0 (0.0)	0 (0.0)	2 (10.0)	1 (6.7)	1 (3.7)	4 (3.1)	0.98
	W	22 (91.7)	44 (97.8)	10 (50.0)	4 (26.7)	2 (7.4)	82 (62.6)	0.81
	X	1 (4.2)	1 (2.2)	8 (40.0)	10 (66.7)	24 (88.9)	44 (33.6)	1.00
11–15	B	0 (0.0)	1 (5.3)	0 (0.0)	0 (0.0)	0 (0.0)	1 (1.3)	0.99
	NG	0 (0.0)	0 (0.0)	1 (8.3)	3 (23.1)	0 (0.0)	4 (5.1)	1.00
	W	16 (100.0)	18 (94.7)	7 (58.3)	6 (46.2)	3 (16.7)	50 (64.1)	0.97
	X	0 (0.0)	0 (0.0)	4 (33.3)	4 (30.8)	15 (83.3)	23 (29.5)	0.97
16–23	B	0 (0.0)	0 (0.0)	1 (7.7)	0 (0.0)	0 (0.0)	1 (1.4)	0.57
	C	0 (0.0)	1 (4.5)	0 (0.0)	0 (0.0)	0 (0.0)	1 (1.4)	0.42
	NG	0 (0.0)	0 (0.0)	1 (7.7)	0 (0.0)	0 (0.0)	1 (1.4)	1.00
	W	17 (100.0)	20 (90.9)	9 (69.2)	7 (50.0)	3 (37.5)	56 (75.7)	0.55
	X	0 (0.0)	1 (4.5)	2 (15.4)	7 (50.0)	5 (62.5)	15 (20.3)	0.83
24–44	NG	0 (0.0)	0 (0.0)	1 (50.0)	1 (25.0)	0 (0.0)	2 (8.7)	1.00
	W	9 (100.0)	5 (100.0)	1 (50.0)	2 (50.0)	1 (33.3)	18 (78.3)	1.00
	X	0 (0.0)	0 (0.0)	0 (0.0)	1 (25.0)	2 (66.7)	3 (13.0)	1.00
45 +	W	5 (100.0)	2 (100.0)	0 (0.0)	3 (75.0)	0 (0.0)	10 (76.9)	0.99
	X	0 (0.0)	0 (0.0)	1 (100.0)	1 (25.0)	1 (100.0)	3 (23.1)	1.00

B, C, W, and X *Neisseria meningitidis* serogroups B, C, W, and X, respectively

*NG* Nongroupable serogroups

**Table 4:** Distribution/trend of *Neisseria meningitidis* serogroups confirmed using polymerase chain reaction by sex

		2016 *N* = 94	2017 *N* = 120	2018 *N* = 59	2019 *N* = 56	2020 *N* = 66	Total *N* = 395	Cochran-Armitage *P*-value
**Sex**	**Serogroups**	N (%)	N (%)	N (%)	N (%)	N (%)	N (%)	
Male	B	0 (0.0)	1 (1.5)	0 (0.0)	0 (0.00	0 (0.0)	1 (0.4)	0.37
	C	1 (1.9)	1 (1.5)	0 (0.0)	0 (0.0)	0 (0.0)	2 (0.9)	0.87
	NG	0 (0.0)	0 (0.0)	4 (11.4)	2 (6.7)	1 (2.5)	7 (3.1)	1.00
	W	50 (96.2)	64 (97.0)	19 (54.3)	18 (60.0)	5 (12.5)	156 (70.0)	0.94
	X	1 (1.9)	0 (0.0)	12 (34.3)	10 (33.3)	34 (85.0)	57 (25.6)	0.86
Female	B	0 (0.0)	0 (0.0)	1 (4.2)	0 (0.0)	0 (0.0)	1 (0.6)	1.00
	NG	0 (0.0)	0 (0.0)	1 (4.2)	3 (11.5)	1 (3.8)	5 (2.9)	1.00
	W	42 (100.0)	52 (96.3)	17 (70.8)	10 (38.5)	5 (19.2)	126 (73.3)	0.76
	X	0 (0.0)	2 (3.7)	5 (20.8)	13 (50.0)	20 (76.9)	40 (23.3)	0.75

B, C, W, and X *Neisseria meningitidis* serogroups B, C, W, and X, respectively

*NG* Nongroupable serogroups

## Results

### Sociodemographic effect and serogroup distribution

A total of 2,426 samples from suspected meningitis cases were tested during the study period. Of the total number tested, 16.3% tested positive for *N. meningitidis.* Almost all cases (98.7%) were from regions traditionally within the country’s meningitis belt. In addition, most recorded cases (56.5%) in this study were men (see Table 1). Regarding age group distribution, approximately one-third (33.2%) of the cases were recorded in the age group 5–10 years, and 17–20% of the cases were recorded in the 11–15, 16–23, and 1–4 years age groups (Table 1).

### Trends in *N. meningitidis* serogroups from January, 2016 to March, 2020

Generally, there was a significant increase in the number of cases of identifiable serogroups in 2020 (66 cases) compared with 2018 (59 cases) and 2019 (56 cases), even though only 3 months of data were considered in 2020. Serotype W was predominant until 2019, followed by serogroup X in 2020. There was a significant upward trend for serogroup X (*P* < 0.01) and downward trend for serogroup W (*P* < 0.01). However, no significant trend was observed in any other serogroup in this study (Fig. 3 and Table 2.

### Trends of *N. meningitidis* serogroups in the various age groups

Although there were changes in the number of cases in the various serogroups across the different age groups over the years under study, no significant upward or downward trend was observed in any of the age groups (see Table 3).

### Trends of *N. meningitidis* serogroups in the two sex groups

Serogroup W was the predominant serogroup in both sexes prior to 2019. However, serogroup X emerged as the predominant serogroup among women in both 2019 and 2020. However, in men, serogroup X only emerged as the predominant serogroup in 2020 (see Table 4). No significant trend was observed in any sex group (Table 4).

## Discussion

This report establishes a 5-year trend of CSM-causing *N. meningitidis* serogroups in Ghana’s meningitis belt. A total of 2,426 suspected patients were tested; of these, 887 patients had meningitis confirmed using positive PCR results at the Tamale Zonal Public Health Reference Laboratory. Among the 887 patients, 395 tested positive for *N. meningitidis.*

Generally, this study found that since the outbreak of serogroup W in Ghana in 2016, cases of meningitis due to *N. meningitidis* had been on a downward trajectory until 2020, when there was a marginal spike in the number of cases resulting from the recent outbreak, increasing from 8.8% in 2019 to 24.4% of all suspected cases tested as of March, 2020. The general downward trend of *N. meningitidis* cases from 2017 to 2019 could be primarily explained by the 2016 massive reactive vaccination campaign with the meningococcal polysaccharide ACW vaccine following the outbreak of serogroup W in 2016 [28]. With this, it was expected that some immunity would be achieved, leading to a reduction in yearly cases of meningitis due to serogroup W. As it constituted a larger percentage of cases due to *N. meningitidis*, the overall number of cases due to *N. meningitidis* is expected to decline. However, the increase in the number of cases in 2020 was due to a recently reported outbreak resulting from a nonvaccine type serogroup (specific to the nonvaccine serogroup).

The study reported significant yearly changes in both serogroups W (*P* < 0.01) and X (*P* < 0.01) over 5 years. Although the serogroup W slightly increased in the number of cases in 2017 compared with that in 2016, it has been on a downward trend since then, accounting for only 15.2% of the total *N. meningitidis* cases in 2020, compared with 96.7% in 2017. However, serogroup X has consistently been on an upward trajectory since 2016, accounting for 81.8% of the total *N. meningitidis* cases in 2020, compared with 1.1% in 2016. This finding by the current study is consistent with a recent 9-year study carried out in 2019 in Niger to establish the epidemiology of bacterial meningitis since the introduction of the meningococcal serogroup A conjugate vaccine. A similar trajectory of the serogroups W and X, as the current study, was established by that study [29].

The downward trend of the serogroup W could be due to the interventions put in place by the Ghana Health Service (GHS) following the outbreak in 2016. There was a reactive vaccination campaign for the meningococcal polysaccharide ACW vaccine in the districts affected by the 2016 *N. meningitidis* serogroup W [28] outbreak; thus, it was expected that population immunity would be achieved, resulting in a reduction in the number of cases due to serogroup W *N. meningitidis* over time. Therefore, the downward trend of serogroup W from 2017 to 2020 is attributable to population immunity from the vaccination campaign and community education on precautionary measures. However, unlike serogroup A, which was eliminated in the country just approximately 2 years after the introduction of the monovalent meningococcal A conjugate vaccine, serogroup W persisted, even 4 years after the vaccination campaign of the polysaccharide meningococcal ACW vaccine, although the coverage rates for both campaigns were the same (98% for the monovalent meningococcal A conjugate vaccine in 2012 and over 98% for the polysaccharide meningococcal ACW vaccine in 2016) [30, 31]. This phenomenon may be explained by the relative effectiveness of the conjugate and polysaccharide vaccines; both have been demonstrated to be effective. However, most polysaccharide vaccines induce hyporesponsiveness and are less immunogenic in children under 2 years of age and are unable to induce immunological memory and affinity maturation in older children and adults compared with conjugate vaccines [32, 33].

Moreover, it must be noted that unlike the meningococcal A vaccine, which has been incorporated into the routine vaccination program in the country since 2016, the polysaccharide meningococcal ACW is still not incorporated into the routine vaccination program. This may have led to the dilution of herd immunity resulting from birth and immigration due to the lack of an ongoing vaccination program. Hence, the persistence of serogroup W declined. The lack of continuous vaccination or its incorporation into the routine vaccination program could also lead to future epidemics resulting from the same serogroup as the immunity of sensitised or vaccinated wanes. Therefore, it is necessary to incorporate it into routine vaccination programs to prevent future epidemics. However, the upward trend of serogroup X may be attributable to the unavailability of a vaccine program coupled with a highly unsensitised or susceptible population. The emergence of serogroup X as the predominant aetiological agent in the wake of declining serogroup W in Ghana also reflects past situations wherein the decline of a previously predominant serogroup is the emergence of a different serogroup as the predominant aetiological agent. For instance, serogroup W emerged in 2012 when serogroup A declined after the introduction of the monovalent meningococcal A conjugate vaccine in Burkina Faso, Mali, and Niger in 2010 [19–21]. Moreover, one study reported a sizeable proportion of serogroup X during the 2017 outbreak in Togo, which constituted 37% of the cases [34]. However, unlike the recent outbreak in Ghana, which this study reports, where serogroup X is the predominant agent (81.1%), it was not the predominant aetiological meningococcal agent in the 2017 outbreak in Togo.

Although there were changes in the number of cases in both serogroups across all age groups and years in this study, there was no significant change in any of the serogroups in any age group. However, consistent with a study carried out in Niger in 2019 (4.2%) [29], cases of *N. meningitidis* were uncommon among children aged under 1 year in the current study (2.0%), and all cases in this age bracket in this study were caused by serogroup W. Except for serogroup B, this study found that cases of all the other serogroups considered were most common among the age group 5–10 years. Overall, cases in this age group (5–10 years) constituted 33% of the *N. meningitidis* in this study. However, 75.8% of the cases in the Niger study were found in the age group 1–14 years [29].

This study’s sex distribution of the serogroups also followed the general trend described in Table 3 in the Results section. However, there was no significant change in any of the serogroups in any of the sex groups.

Although there is currently no licenced vaccine targeting serogroup X in the country and region as a whole, a pentavalent meningococcal conjugate vaccine (MenACWXY) has been developed and is currently in the later phase of clinical trials [35]. Therefore, it is recommended that the country positions itself well by identifying sustainable funding sources so that when the vaccine becomes available, it is procured immediately. However, in the interim, case-based surveillance and community sensitisation should be intensified across all regions within the country’s meningitis belt. It is also recommended that robust surveillance be instituted by the GHS for all other nonvaccine types and NG serogroups so that an emerging threat is identified from the onset. The capacity for testing should also be built for all regions within the country’s ‘meningitis belt’ to aid rapid testing. The molecular epidemiology of serogroup X is recommended to determine whether the circulating strains are the same or different. This will aid in the development of an appropriate targeted approach.

It is important to note that the study was limited by the number of months of cases in 2020 included in the analysis due to the coronavirus outbreak. This was limited by the lack of completeness of information related to some cases, resulting in their exclusion from the study.

## Conclusion

The results of the analysis of cases of bacterial meningitis due to *N. meningitidis* from 2016 to 2020 show the emergence of serogroup X, a nonvaccine type. After the mass reactive vaccination campaign of the meningococcal polysaccharide ACW vaccine following the outbreak of serogroup W in the upper west region in 2016, its prevalence has declined, paving the way for serogroup X as the predominant serogroup. However, unlike serogroup A, which had not been recorded since 2014 after the introduction of the meningococcal A conjugate vaccine in 2012, serogroup W persisted, even 4 years after the meningococcal ACW polysaccharide vaccine mass vaccination campaign.

Based on the findings and conclusions drawn from this study, it is recommended that sustainable funding sources be identified so that when the pentavalent meningococcal vaccine, MenACWXY, becomes available, it is procured for immediate use. It is also recommended that the polysaccharide meningococcal vaccine, ACW, be incorporated into routine vaccination programs, and that there should be an intensification of case-based surveillance and community sensitisation across all regions within the country’s meningitis belt. The immunogenicity of polysaccharide meningococcal vaccine, ACW, also needs to be studied, especially with prior or concomitant immunisation with other vaccines, such as tetanus-containing vaccines, which have been shown to influence the immunogenicity of some meningococcal vaccines [36]. An institution of strong surveillance for all the other serogroups, including the NG ones, and establishing testing centres in all the regions within the meningitis belt are recommended to identify emerging threats from the onset and to ensure immediate testing once a case suspicion is made. Finally, there is also the need to characterise all serogroup X cases for all years to determine whether the same strain is in circulation or whether there are varying strains.

## Supplementary Information

**Additional file 1.**

## Data Availability

The datasets used and/or analysed during the current study are available from the corresponding author upon reasonable request. However, the minimal dataset analysed during this study is included in this article (and its Supplementary information files) in the Supporting Information section.

## Abbreviations

CSM: Cerebrospinal meningitis
PCR: Polymerase chain reaction
NG: Nongroupable
CSF: Cerebrospinal fluid
GHS: Ghana health service
